# Valorization of Winery By-Products as Bio-Fillers for Biopolymer-Based Composites

**DOI:** 10.3390/polym16101344

**Published:** 2024-05-09

**Authors:** Filippo Biagi, Alberto Giubilini, Paolo Veronesi, Giovanni Nigro, Massimo Messori

**Affiliations:** 1 Department of Civil, Chemical, Environmental, and Materials Engineering (DICAM), University of Bologna, Via Zamboni 33, 40126 Bologna, Italy; filippo.biagi3@unibo.it; 2 Department of Management and Production Engineering (DIGEP), Politecnico di Torino, Corso Duca degli Abruzzi 24, 10129 Torino, Italy; 3 Department of Engineering “Enzo Ferrari” (DIEF), University of Modena and Reggio Emilia, Via Pietro Vivarelli 10, 41125 Modena, Italy; paolo.veronesi@unimore.it; 4 Ri.Nova—Filiera Vitivinicola ed Olivo-Oleicola, Via Tebano 45, 48018 Faenza, Italy; gnigro@rinova.eu; 5 Department of Applied Science and Technology (DISAT), Politecnico di Torino, Corso Duca degli Abruzzi 24, 10129 Torino, Italy; massimo.messori@polito.it

**Keywords:** bio-composites, melt-compounding, biopolymers, grape seeds, wine lees, grape pomace

## Abstract

Grape seeds (GS), wine lees (WL), and grape pomace (GP) are common winery by-products, used as bio-fillers in this research with two distinct biopolymer matrices—poly(butylene adipate-*co*-terephthalate) (PBAT) and polybutylene succinate (PBS)—to create fully bio-based composite materials. Each composite included at least 30 v% bio-filler, with a sample reaching 40 v%, as we sought to determine a composition that could be economically and environmentally effective as a substitute for a pure biopolymer matrix. The compounding process employed a twin-screw extruder followed by an injection molding procedure to fabricate the specimens. An acetylation treatment assessed the specimen’s efficacy in enhancing matrix–bio-filler affinity, particularly for WL and GS. The fabricated bio-composites underwent an accurate characterization, revealing no alteration in thermal properties after compounding with bio-fillers. Moreover, hygroscopic measurements indicated increased water-affinity in bio-composites compared to neat biopolymer, most significantly with GP, which exhibited a 7-fold increase. Both tensile and dynamic mechanical tests demonstrated that bio-fillers not only preserved, but significantly enhanced, the stiffness of the neat biopolymer across all samples. In this regard, the most promising results were achieved with the PBAT and acetylated GS sample, showing a 162% relative increase in Young’s modulus, and the PBS and WL sample, which exhibited the highest absolute values of Young’s modulus and storage modulus, even at high temperatures. These findings underscore the scientific importance of exploring the interaction between bio-fillers derived from winery by-products and three different biopolymer matrices, showcasing their potential for sustainable material development, and advancing polymer science and bio-sourced material processing. From a practical standpoint, the study highlighted the tangible benefits of using by-product bio-fillers, including cost savings, waste reduction, and environmental advantages, thus paving the way for greener and more economically viable material production practices.

## 1. Introduction

In recent decades, driven by increasing environmental concerns, both industry and scientific researchers have shifted their focus towards developing new processes, materials, and systems with reduced impacts [1,2]. Within the world of plastics, biopolymers, derived from renewable sources (bio-based) and/or biodegradable ones, have gained great attention as promising solutions for reducing dependence on fossil fuels and mitigating plastic pollution [3,4,5].

In this context, poly(butylene adipate-*co*-terephthalate) (PBAT), also known as polybutyrate, is a semi-aromatic, biodegradable thermoplastic copolyester, which is primarily derived from fossil sources, but holds potential for use in more environmentally sustainable production methods [6,7]. It comprises two distinct repeating units, butylene terephthalate (BT) and butylene adipate (BA), bonded through a condensation reaction, with their molar ratios influencing the copolymer’s properties [8]. Another promising biopolymer that represents a valid alternative to fossil-based polymers is polybutylene succinate (PBS). It is a semicrystalline thermoplastic polymer belonging to the family of aliphatic polyesters, characterized by its excellent biodegradability, processability, and mechanical properties comparable to traditional polymers such as polypropylene (PP). PBS is synthesized by the poly-condensation of 1,4-butanediol and succinic acid. It is generally produced from fossil sources but is gradually being derived from renewable resources. Nowadays, these monomers have also begun to be obtained through the fermentation of agro-industrial wastes or other renewable sources, and the biomass-derived content of PBS is estimated to represent 80% [9].

Despite their promising attributes, biopolymers cover only a limited portion of the market due to their higher costs compared to conventional polymers. In 2018, the global production of bio-based/non-biodegradable and biodegradable polymers amounted to 1.2 million tons and 0.91 million tons, respectively, representing only 0.35% and 0.26% of total polymer production [9]. Nonetheless, the biopolymer market is constantly expanding, with bio-based alternatives becoming increasingly available across various sectors, from agri-food to biomedical applications [10]. A common contemporary research field is interested in developing new reinforced and bio-based composites starting from polymer matrix and natural fillers, due to their advantages such as low density and low cost, as well as being environmentally friendly and biodegradable [11,12,13,14,15].

One interesting example of a bio-filler application in composite materials is in the civil and construction sector, where the demand for eco-friendly construction materials has led to growing interest in incorporating plant-based fibers into cement composites. These fibers, derived from renewable sources, offer cost-effectiveness and additional benefits including lightweight properties, enhanced stiffness, and exceptional resilience to impacts. For example, Laverde et al. conducted a comprehensive review of various natural fibers, such as sisal, flax, and coconut, to be used in a cement–matrix composite material, treated in such a way as to enhance the final mechanical properties [16]. Regarding polymer matrices, several natural-based fillers have been studied. For example, previous studies explored the effects of grape leaf fibers used as reinforcing agents for high-density polyethylene (HDPE) on both thermal stability and mechanical performance [17]. However, despite the potential benefits of using this bio-sourced filler, the composites exhibited an overall reduction in both thermal stability and mechanical properties. Another study explored a polymer blend comprising poly(vinyl alcohol) (PVA), chitosan, and grape seeds extract. This investigation highlighted the promising potential of the composite as a green alternative for use in flexible electronic, electrochemical, and energy storage devices, thanks to the exceptional mechanical, thermal, and electrical properties it demonstrated [18]. PBAT and PBS are two biopolymers widely used in the literature as matrices for different bio-fillers, thanks to their excellent and wide processability, including injection molding, extrusion, and blow molding. A significant example was seen in the investigation of mangosteen and durian peel waste as bio-fillers and natural pigments for PBAT composites [19]. Additionally, agar was used as a bio-filler with PBAT matrix to tune the chemical and physical properties of the biodegradable composite across different filler percentages [20]. In another previous study, the biodegradation of PBS–wheat bran composites was investigated [21].

An important aspect of the use of bio-fillers, which has often been overlooked in previous literature, is the quantity of the available bio-fillers, which may or may not support production volumes at an industrial level, and thus may be exclusively oriented towards use in laboratory- or small-scale applications. Considering the substantial volume of winery by-products generated annually, with global grape production estimated at approximately 24.5 million metric tons in 2021 [22], and up to 2800 tons in just one province of northern Italy [23], winery by-products represent a promising bio-filler option for investigation. The primary by-products form the winery production chain are wine lees (WL), grape pomace (GP) and grape seeds (GS) [24]. WL consists of both solid and liquid phases containing various organic substances. GP is the most abundant by-product of the wine-making industry [22], and it is composed of cellulose, hemicellulose, lignin, proteins, soluble polyphenols, ashes, condensed tannins, pectin substances, and neutral polysaccharides. GS primarily consists of dietary fibers, proteins, lipids, polyphenolic compounds, and carbohydrates [14].

In this work, these three winery by-products were investigated as potential bio-fillers for melt compounding with PBAT and PBS matrices to produce cheap and environmentally friendly final composites, potentially on a large scale. A solvent-free acetylation treatment was also evaluated to enhance the matrix–filler interaction. The structural, mechanical, thermal, and hygroscopic properties were measured to assess the potential uses of these bio-composites in various possible fields of application, including reemployment in the wine industry. Therefore, this study holds dual significance. From a scientific perspective, it aims to advance our understanding of the interaction between three different bio-fillers and two biopolymer matrices, representing systems never before explored in these specific combinations, to the authors’ knowledge. On a practical level, the study seeks to produce and evaluate winery by-product powders as bio-fillers for bio-composites production. This approach could not only reduce the cost of the final products, by decreasing the amount of biopolymer matrix, but could also valorize industrial waste, thereby minimizing accumulation in landfills or soil, thus preventing potential phytotoxicity issues in the microbiota and the environment [25].

## 2. Materials and Methods

**Figure 1:**
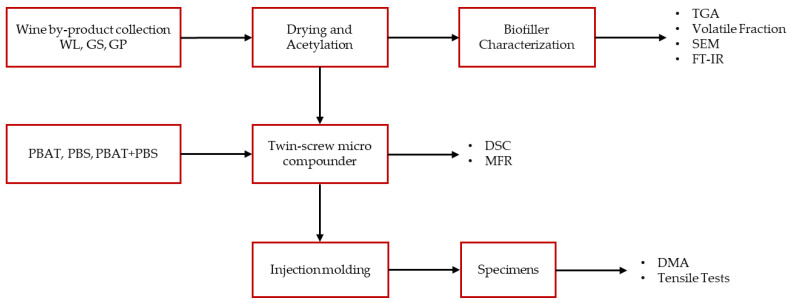
Schematic representation of the fabrication and characterization of the bio-composite samples.

Figure 1 illustrates a schematic representation of the overall structure adopted in this research article, starting from the collection of raw materials, their processing, and finally the characterization of the bio-composites. The following subsections provide a detailed presentation of the processing conditions and characterization techniques employed.

### 2.1. Materials

Poly(butylene adipate-*co*-terephthalate) (PBAT) and poly(butylene succinate) (PBS) were purchased from NaturePlast (NaturePlast, Caen, France) in pellet form and the properties declared in the supplier’s technical data sheets are presented in Table 1. Three different bio-fillers—wine lees (WL), grape pomace (GP) and grape seeds (GS)—were provided by the winery Cevico Group C.V.C (Cevico Group C.V.C., Lugo, Italy), derived from red Sangiovese and Cabernet grapes. All bio-fillers underwent an initial drying process at 70 °C until reaching a constant mass. Subsequently, they were ground using an A 10 basic mill (IKA, Staufen, Germany) and finally sieved through a 1 mm mesh size sieve.

### 2.2. Wine By-Products’ Functionalization

The acetylation process was conducted in accordance with the methodology outlined in prior research by Olaru et al. [26]. This approach stands out from most acetylation reactions documented in the literature due to its solvent-free nature. The functionalization was carried out exclusively on WL and GS. First, acetic anhydride (85 mL) was mixed with sulfuric acid (170 μL) to serve as a catalyst, and then the ground bio-filler (17 g) was added to this solution under magnetic stirring. The acetylation reaction was performed in a round-bottom flask immersed in a water bath at 30 °C for 6 h. Subsequently, samples were washed by centrifugation (7500 rpm for 5 min) and redispersion, first with water, then with water:ethanol (1:1) solution, and finally with pure ethanol. Lastly, the samples were dried overnight at 70 °C.

### 2.3. Bio-Filler Characterization

#### 2.3.1. Volatile and Not-Volatile Solid Fractions

The volatile ($S_{v}$) and not-volatile ($S_{nv}$) solid fractions were quantified using a muffle furnace operating at 650 °C for 2 h, employing Equations (1) and (2):

(1) $$S_{nv}\left(\%\right)=\frac{W_{650}}{W_{80}}\times 100$$

(2) $$S_{v}\left(\%\right)=\frac{{W_{80}-W}_{650}}{W_{80}}\times 100$$

where $W_{650}$ and $W_{80}$ represent the weights of the residues at 650 and 80 °C, respectively.

#### 2.3.2. Fourier-Transform Infrared Spectroscopy (FT-IR)

Infrared spectra of dried unmodified and acetylated WL and GS were recorded on a Nicolet iS 50 Spectrometer (Thermo Scientific, Waltham, MA, USA). The pre- and post-functionalization spectra were recorded between 4000 and 500 cm^−1^, with a resolution of 4 cm^−1^ and 32 scans, using the Omnic software from Thermo Fischer Scientific.

#### 2.3.3. Granulometry

The particle size distribution was evaluated on a Mastersizer 3000 (Malvern Panalytical, Malvern, UK).

#### 2.3.4. Density Pycnometry

The true density (ρ) of the powders was determined using a helium pycnometer UltraPyc 5000 (Anton Paar Italia S.r.l, Rivoli, Torino, Italy). Approximately 10 g of sample was introduced into the 54 cm^3^ sample chamber, and the density was measured with a minimum of 3 passes, each consisting of 3 measurements, to ensure a final level of 0.05% accuracy.

#### 2.3.5. Scanning Electron Microscopy (SEM)

The morphology of all types of bio-fillers was investigated using a Phenom™ XL G2 Desktop SEM (Thermo Fisher Scientific, Waltham, MA, USA) at an accelerating voltage of 15 kV. Previously, each specimen was mounted on a carbon tape and sputter-coated with a layer of gold for 3 min at 10^−3^ mbar and 10 mA current flow (SPI Supplies, Complete Sputter Coating System, West Chester, PA, USA).

#### 2.3.6. Thermogravimetric Analysis (TGA)

TGA measurements were conducted on a TGA/SDTA 851 instrument (Mettler-Toledo, Greifensee, Switzerland), equipped with a microbalance with a precision of ±0.1 mg. Specimens weighing 12 ± 2 mg were placed into 70 mL alumina crucibles. To characterize the thermal stability, the specimens underwent heating from 25 to 900 °C at a rate of 10 °C/min, while maintaining a flow rate of 50 mL/min in both air and nitrogen atmospheres.

### 2.4. PBAT/PBS-Based Composites Preparation

Different bio-composites were prepared via melt extrusion, with sample codes and compositions detailed in Table 2. Prior to extrusion, 1 wt. % of paraffin oil (relative to the total extruded mass) was manually incorporated to enhance the wetting and adhesion between the polymer and the bio-fillers, thereby improving surface adherence. All bio-composites were processed using a twin-screw micro compounder with a batch volume of 15 cm^3^ and conical screws, operating at a processing temperature of 130 °C, a mixing speed of 100 rpm, and a mixing time of 2 min.

After compounding, the resultant samples underwent grinding and were then processed via injection molding (TECNICADUEBI MegaTech H7/18–1, Fabriano, Italy) to produce standard tensile test specimens, conforming to the UNI EN ISO 527-2 (type 1 BA) standard [27]. Four characteristic temperatures were set in this manufacturing process: specifically, a plasticizing temperature of 140 °C, an injection temperature of 135 °C, a nozzle temperature of 135 °C and a mold temperature of 22 °C. In addition, an injection pressure of 120 bar, a holding pressure of 40 bar, a holding time of 3 s, as well as a cooling time of 6 s, were set.

### 2.5. PBAT/PBS-Based Composites Characterization

#### 2.5.1. Differential Scanning Calorimetry (DSC)

The thermal properties of the bio-composite samples were assessed using a PerkinElmer Pyris 1 instrument (Shelton, CT, USA) connected to a cooling system Intracooler 2P. Each specimen, weighing 10 ± 2 mg, was analyzed under nitrogen purging at a flow rate of 20 mL/min. The heating protocol involved initial heating from 40 to 180 °C at 15 °C/min for PBAT-based composites and from 40 to 200 °C at 15 °C/min for PBAT + PBS-based and PBS-based composites, aimed at erasing the previous thermal history. Subsequently, samples were cooled to −40 °C at 10 °C/min for all compositions. Finally, they were reheated to 180 °C at 10 °C/min for PBAT-based materials and to 200 °C at 10 °C/min for PBAT + PBS-based and PBS-based composites. During the cooling cycle, crystallization temperature ($T_{c}$) and crystallization enthalpy ($H_{c}$) were determined, while during the second heating cycle, melting temperature ($T_{m}$), melting enthalpy ($H_{m}$) and glass transition temperature ($T_{g}$) were measured. Melting enthalpies were calculated by considering the additives’ weight fractions. The crystallinity degree ($X_{c}$) was calculated using the Equation (3):

(3) $$X_{c}=\left(\frac{H_{m}-H_{cc}}{H_{ref}\times (1-w_{add})\;}\right)\times 100$$

where $H_{ref}$ represents the reference melting enthalpy of the 100% crystalline polymer and $w_{add}$ the weight fraction of the different bio-fillers (WL, GP, GS). The reference melting enthalpy values were set as 114 J/g for PBAT [28] and 110 J/g for PBS [29]. For PBAT + PBS-based composites, the values considered were 112 J/g for the 50%-50% composition and 67.2 J/g for the 30%-30% compositions.

#### 2.5.2. Hygroscopic Analysis

To determine the hygroscopic properties of the composites, a climatic chamber CLIMATEST CH150 (ARGOLab, Modena, Italy) was set at a temperature of 20 °C and a relative humidity of 60%. Rectangular specimens measuring 33 × 9 × 1.8 mm^3^ were weighed at intervals of 24, 48, 120, 144, 168, and 216 h.

#### 2.5.3. Mass Melt Flow Rate (MFR)

The polymer MFR was measured following the ASTM D 1238 standard procedure. A 5 g sample, in pellet form, was pre-heated for 5 min within the barrel and subsequently extruded through the die under a steady 2.16 kg load. This assessment of the MFR was conducted for all compositions at a temperature of 190 °C.

#### 2.5.4. Dynamic Mechanical Analysis (DMA)

The dynamic mechanical behaviors of all samples were evaluated using a DMTA 3E instrument (TA Instruments, New Castle, DE, USA) in the dual cantilever bending geometry configuration. Rectangular specimens measuring 33 × 9 × 1.8 mm^3^ were used for DMA tests. The analysis was conducted with a heating rate of 2 °C/min from −20 to 80 °C, while maintaining an oscillation frequency of 1 Hz and a strain of 0.1%. For each sample, the storage modulus (E’) was plotted as a function of temperature.

#### 2.5.5. Tensile Tests

Tensile tests were conducted using an Instron 5966 machine (Instron, Norwood, MA, USA) equipped with a 10 kN load cell. A crosshead speed of 50 mm/min was used for analysis. The tests were conducted on injection-molded specimens of neat biopolymers and bio-composites.

## 3. Results and Discussions

### 3.1. Bio-Fillers Characterization

**Figure 2:**
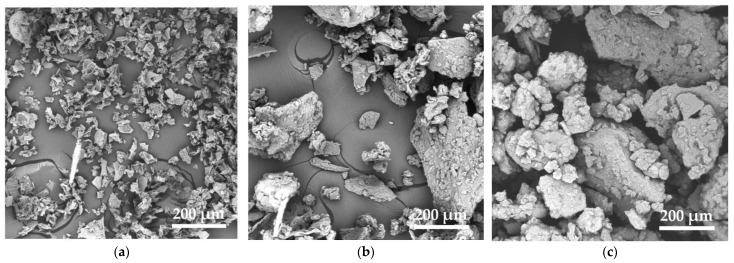
SEM micrographs of three different bio-fillers: (**a**) wine lees (WL), (**b**) grape pomace (GP) and (**c**) grape seeds (GS).

A morphological characterization of the bio-fillers was performed to determine their dimensions and shapes, and the main findings are depicted in Figure 2. All bio-filler powders possess a flake-like shape, with WL being notably smaller in size compared to the other two fillers, which conversely share similar shapes and sizes. This discrepancy could arise from the notable presence of both linoleic and oleic acids [30] in the wine seeds, which may lead to particle aggregation.

**Figure 3:**
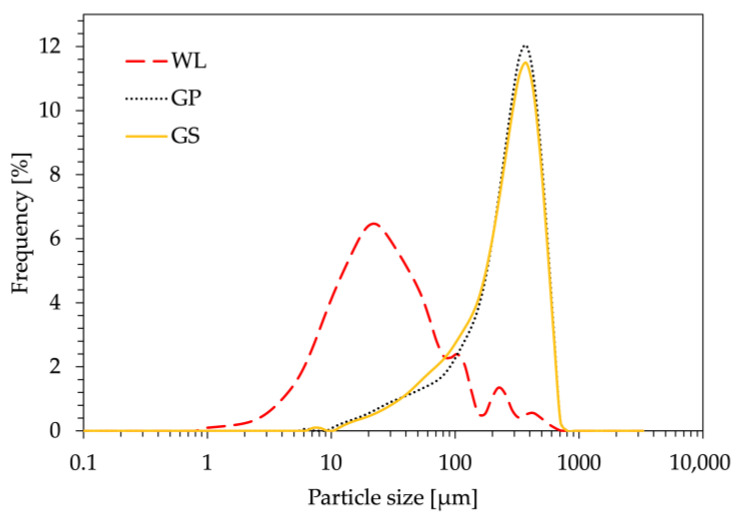
Granulometric analysis of three different bio-fillers derived from wine by-products: wine lees (WL), grape pomace (GP) and grape seeds (GS).

These morphological results were also confirmed by the granulometric analysis, the results of which are presented in Figure 3.

It is evident that all particle size distribution curves exhibit a Gaussian pattern; however, the individual values of D10, D50, and D90 vary significantly, which is in good agreement with the SEM micrographs. Notably, the D10 and D50 values of WL particles are one order of magnitude smaller than those of GP and GS particles. The comprehensive set of values, including the real densities (ρ) calculated for all three powders through gas pycnometry, is listed in Table 3. This difference in dimension implies potentially superior final mechanical performance in bio-composites with WL compared to those with GP and GS. The larger dimensions of the latter may act as stress concentrators, thereby causing a reduction in tensile strength, a well-established phenomenon documented in prior literature [31,32].

**Figure 4:**
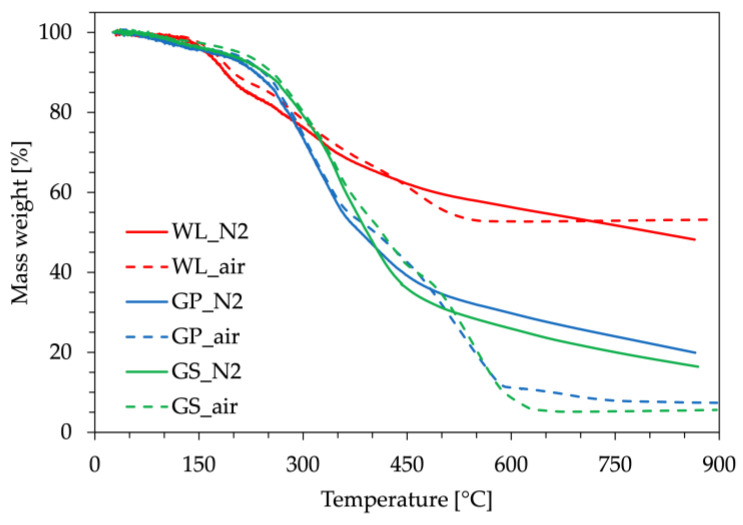
Thermogravimetric curves of three different bio-fillers: wine lees (WL), grape pomace (GP) and grape seeds (GS) in two distinct conditions: inert (N2) and oxidizing (air) atmospheres.

Due to the crucial role that thermal stability plays during the further processing of bio-fillers and final bio-composites, thermogravimetric analyses were carried out to gain deeper insights into the thermal behavior of the bio-fillers and evaluate their volatile fraction. Figure 4 and Table 4 summarize the main findings obtained by these characterizations.

Figure 4 illustrates the TGA curves for all three of the bio-fillers under two distinct atmospheres, nitrogen and air. All bio-fillers demonstrated a thermal stability that makes them suitable for further processing with PBAT and PBS, without the risk of degradation during extrusion and injection molding procedures. However, it can be observed that WL initiates thermal degradation at lower temperatures, compared to the other two bio-fillers. For example, considering a mass loss of 10 wt. %, the temperature for WL is approximately 44 °C and 56 °C lower than that of GP and GS, respectively. Moreover, the bio-filler residues at 900 °C under nitrogen atmosphere are notably higher for WL (48 wt. %) compared to GP (20 wt. %) and GS (16 wt. %). Under an oxidizing atmosphere, both GP and GS exhibit residues of about 7 wt. %, whereas no significant variation is observed for WL. This gap may be attributed to the combustion in the presence of oxygen of the organic additives, resulting in greater weight loss. This finding suggests that GP and GS possess a higher organic content compared to WL; this was also confirmed by the muffle-furnace test, which detected higher inorganic and not-volatile fractions in WL with respect to GP and GS (Table 4). These results are consistent with prior published literature [15]. The presence of inorganic components such as potassium, calcium, or silicon could be attributed to the presence of tartrate salts derived from the significant tartaric acid content often found in WL [33,34], or contamination with kaolin or bentonite, which are commonly used in wine clarification treatments [35]. Also in this case, the high inorganic content of WL suggests its potential use as a bio-filler despite being an agro-industrial by-product.

**Figure 5:**
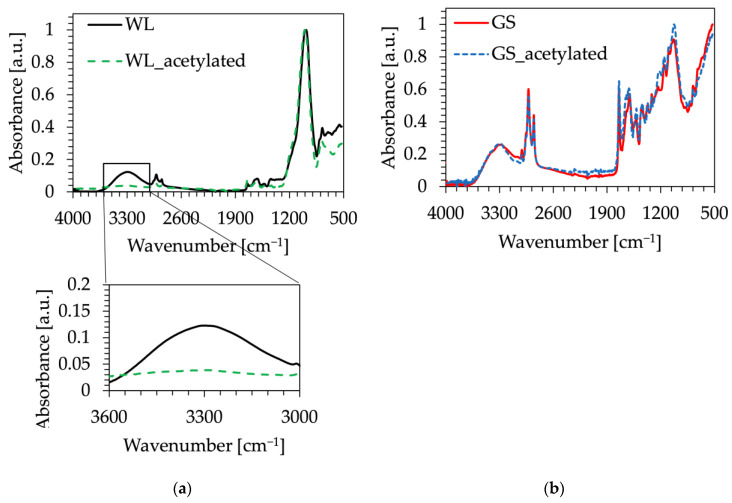
FT-IR spectra of: (**a**) wine lees (WL) and acetylated wine lees (WL_acetylated) powder; (**b**) grape seeds (GS) and acetylated grape seeds (GS_acetylated) powder.

To evaluate the effects of the acetylation procedure over WL and GS, FT-IR analysis was conducted on untreated and functionalized powders (Figure 5a,b). No significant variations were found between the two powders analyzed, except for a reduction in the hydroxyl band in WL powder, around 3300 cm^−1^, which could be attributed to the substitution of hydroxyl groups by acetyl groups (Figure 5a), as was also observed in previous research [36].

### 3.2. Bio-Composites Characterization

#### 3.2.1. Thermal and Viscosity Characterization

The first characterization performed on the final bio-composites involved examining their main thermal properties, as detailed in Table 5 alongside MFR values.

The PBAT-based composites showed large melting peaks due to the low crystallinity percentages of these composites. In PBS-based composites, the bio-fillers slightly slowed down the crystallization process by interfering with the PBS polymer chains. In fact, both the *T_c_* and *T_m_* of the neat biopolymer (C0) were higher compared to those of the bio-composites (C1, C2, C3, C4, C5). However, aside from this minor aspect, no other significant changes in the thermal properties of the bio-composites were observed, indicating that the addition of bio-fillers did not affect the thermal properties of the starting biopolymers. These findings align with previous literature on the subject [37,38,39]. Typically, when the particle size of fillers is small enough, they can function as nucleating agents, thereby promoting the formation of crystalline domains [40,41,42]. However, in this instance, the crystallinity of bio-composites was not influenced by the addition of bio-fillers. This unaltered crystallinity may indicate the need for the further optimization of the grinding process to enhance the crystal domains. Nevertheless, the bio-fillers were not excessively coarse, as evidenced by the preservation of crystallinity. Indeed, the use of overly large fillers can lead to agglomeration phenomena, which may hinder crystallization [15]. Besides this, all the bio-fillers slightly decreased the viscosity of PBAT and PBS-based composites, as indicated by the MFR values, similarly to what was observed in other previous studies with PBAT and other bio-filler, such as almond shell [43] or biochar [44]. According to the smallest dimensions exhibited by WL, the MFR values of their bio-composites are the highest among all three compositions. For all samples, the decrease in viscosity did not affect the processability of the injection molding process.

#### 3.2.2. Hygroscopic Gravimetric Analysis

**Figure 6:**
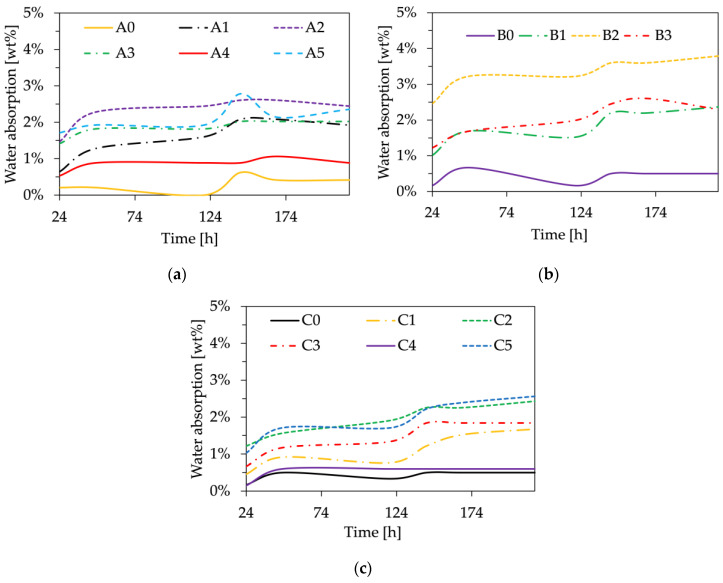
Hygroscopic behavior of bio-composites: (**a**) PBAT-based composites; (**b**) PBAT/PBS-based composites; (**c**) PBS-based composites.

The addition of the bio-fillers in the polymer matrix caused an increase in the water absorption of the material, as can be seen from Figure 6. This effect can be attributed to the fact that natural fillers increase both hydrophilicity [45,46] and porosity [46], with respect to the neat hydrophobic biopolymer, thereby facilitating the adsorption and transport of water from surface to polymer bulk. The bio-composites containing WL (A1 and C1) exhibited lower water absorption compared to those containing GP and GS (A2, A3, C2, C3), mainly due to the smaller sizes of the particles. In addition, it is noteworthy that the acetylation treatment of WL decreased its hygroscopic tendency by improving the affinity between the filler and the polymer matrix, as well as the surface roughness of the samples. This observation aligns well with findings from previous FTIR analyses, where the -OH band showed a significant reduction, indicating a decrease in hydroxyl groups. Furthermore, the GP bio-composites (A2, B2, C2) displayed the highest degree of hygroscopicity, both after 24 h and at the test’s conclusion, suggesting that this filler possesses the greatest affinity for water. During the test, as depicted in the graphs in Figure 6, some mass fluctuations were observed, and they may be ascribed to the release of CO_2_ from the samples.

#### 3.2.3. Tensile Properties

After conducting thermal and water affinity characterization, mechanical investigation was carried out via tensile testing of the bio-composites exclusively for samples containing acetylated bio-fillers. This decision was made under the assumption that the functionalization process enhanced the interaction between the additive and biopolymer matrix, consequently improving the mechanical performance. Table 6 presents the main results regarding Young’s modulus (E), ultimate tensile strength (UTS), and elongation at break (ε_b_). It is important to note a significant stiffening effect due to the addition of bio-fillers (A4, A5, C4, C5) compared to the neat biopolymer matrices (A0, C0). Specifically, acetylated WL samples (A4, C4) exhibited increases in Young’s modulus of approximately 110% and 65%, respectively, compared to the neat polymers (A0, C0). This effect is even more pronounced in the case of composites with GS (A5, C5), where the increase in stiffness reached 162% and 67% compared to the biopolymer matrices (A0, C0).

The increment in Young’s modulus can be attributed to the higher inherent stiffness of WL and GS particles, due to the substantial inorganic fractions, rather than the polymer matrices. However, considering UTS and elongation at break, all the bio-composites (A4, A5, C4, C5) exhibited worse properties than the neat polymers (A0, C0), indicating a need for improvement in the stress transmission mechanism between the matrix and fillers. The trends of E, UTS and ε_b_ are consistent with those in the previous literature [34]. Hence, future studies should concentrate on optimizing the functionalization process to enhance compatibility between the matrix and the bio-filler. While a direct comparison with results from previous studies on the same biopolymer matrices with different bio-fillers is not feasible, it is still insightful to draw connections that will underscore the advantages of our matrix–filler system over samples previously investigated in the literature. For instance, it is noteworthy that our PBAT/acetylated WL bio-composite (A4) exhibited a Young’s modulus that is potentially double that achieved by specimens filled with biochar, at a concentration of 20 wt. % [44]. Similar observations can be extended to PBS-based composites as well. For example, in our case study, grape seeds exerted a more pronounced stiffening effect compared to almond shell flour, as reported by Limiñana et al. [47].

#### 3.2.4. Dynamic Mechanical Analysis (DMA)

**Figure 7:**
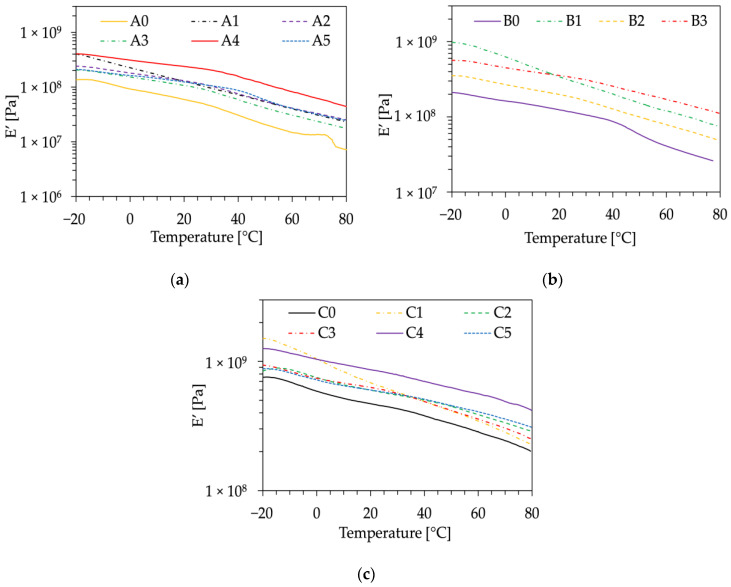
Storage moduli (E’) as a function of temperature of: (**a**) PBAT-based composites; (**b**) PBAT/PBS-based composites; (**c**) PBS-based composites.

To conduct a comprehensive mechanical analysis of the composites, DMA was performed on all samples. The storage modulus (E’) as a function of temperature, ranging from −20 to 80 °C, is illustrated in Figure 7, and significant values at specific temperatures are listed in Table 7.

The storage modulus of the neat biopolymer was consistently lower than that of the bio-composite due to the higher stiffness of the fillers, confirming observations derived from tensile testing. The samples exhibiting the highest E’ were those with PBS as the matrix and WL as the bio-filler, in both untreated and acetylated compositions, namely, samples C1 and C4. This could be ascribed to the stiffening effect imparted by the rigid inorganic particles with a low diameter characteristic of WL, which is in good agreement with all previously presented findings. Notably, at higher temperatures such as 50 °C or 75 °C, the effect of acetylation on WL became more pronounced. In fact, the E’ for acetylated WL is almost always double that of the bio-composites containing untreated WL (A1, C1). Regarding the bio-composites with GP as bio-filler (A2, B2, C2), they exhibited the lowest E’ values, probably due to the low cellulose and hemicellulose content. These results are consistent with previously published literature [9,37].

**Figure 8:**
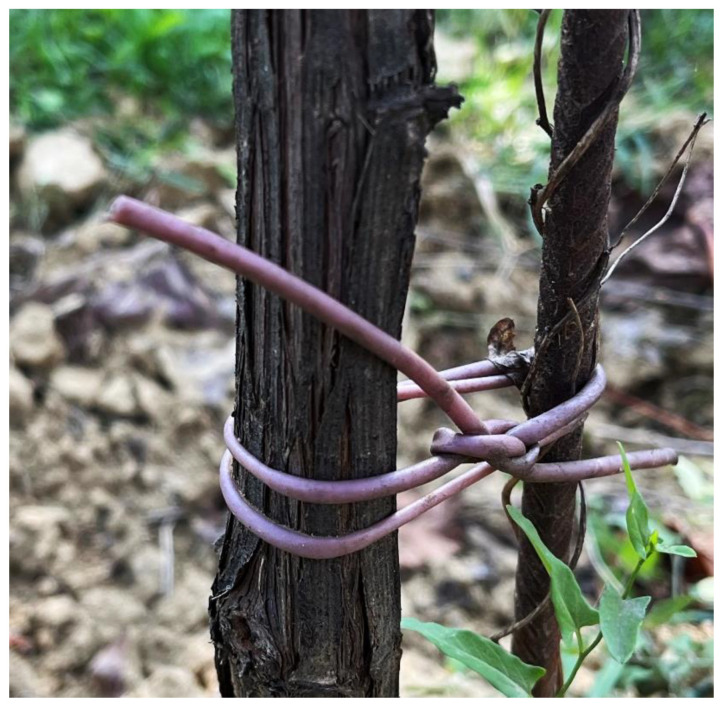
Example of application of a PBAT-based and wine lees composite.

The results presented in this preliminary study on these novel bio-composites are both interesting and promising. Starting from the characterization of the bio-filler powders, which has often been overlooked in previously published studies, it is evident that all three winery by-products exhibited dimensions, thermal stabilities, and compositions that made them suitable as bio-fillers. Among these, WL appeared to be the most promising in terms of its ability to act as a reinforcing agent, and not only as a filler, especially in its acetylated form. The two biopolymer matrices have very different initial properties, and for this reason, it is plausible to use them for different final applications. For example, PBS-based composites might be of interest in applications where properties of greater stiffness are desired, while PBAT-based bio-composites would be useful for end-usable parts requiring higher flexibility. For example, PBAT-WL composites were used to create biodegradable vineyard ties (Figure 8), offering sustainable substitutions for conventional PVC ties, providing structural support to grapevines while mitigating environmental impacts.

Future research developments may be oriented towards a deeper characterization of the bio-composites, including assessing their actual biodegradation and the effects of bio-fillers on degradation rates. Additionally, evaluating the sustainability and economic viability of the acetylation process would be beneficial.

## 4. Conclusions

In this study, bio-composites were developed using neat PBAT, neat PBS, and a PBAT/PBS blend as matrices, combined with winery by-products such as wine lees, grape seeds, and grape pomace serving as bio-fillers. These bio-composites underwent processing through twin-screw extrusion and injection molding, followed by comprehensive characterization. The main conclusions drawn from these findings are as follows:

1. The incorporation of bio-fillers did not significantly alter the thermal properties of the resulting composites, ensuring their stability across varying temperature conditions;
2. The compounding of bio-fillers decreased the viscosity of the composites, most significantly for WL, probably due to its inherent dimensions, without compromising the processability of the bio-composites;
3. All bio-composites exhibited increased water affinity, with GS-based composites showing the highest affinity. Acetylated samples demonstrated slightly lower water absorption, attributed to reduced hydrophilicity;
4. Tensile testing revealed a noticeable stiffening effect in the bio-composites, especially those filled with WL. The Young’s modulus reached approximately 1.5 GPa, with PBS matrices exhibiting a higher stiffening effect compared to PBAT. Specifically, in PBAT-based composites, there was a 108% improvement with the addition of WL and a 162% improvement with acetylated WL, while in the case of PBS-based composites, there was an approximately 65% increase in Young’s modulus with both WL and acetylated WL;
5. The stiffening effects of the three different bio-fillers were further evidenced by an increase in the storage modulus across a wide temperature range, highlighting the positive effect of acetylation at higher temperatures.

The findings underscore the potential for the future substitution of traditional petroleum-based polymers with these economically viable bio-composites. This solution not only addresses winemaking waste disposal concerns, but also aligns with the growing demand for environmentally friendly products, thus suggesting a viable mode of valorization of a by-product of the wine production chain. Given the abundant supply, cost-effectiveness, renewable nature, and remarkable versatility of these bio-fillers, wine by-products exhibit considerable potential for widespread use in large-scale applications requiring materials with both a biological origin and specific mechanical properties.

## Figures and Tables

**Table 1:** Properties of biopolymers from supplier’s data sheet.

Biopolymer	Supplier Code	Melting Temperature (°C)	Density (g/cm^3^)	Melt Flow Index (190 °C; 2.16 kg) (g/10 min)
**PBAT**	PBE 006	110–115	1.26	4–6
**PBS**	PBE 003	115	1.26	4–6

**Table 2:** PBAT/PBS-based composite formulations, with different acetylated (Ac.) and neat bio-fillers: wine lees (WL), grape pomace (GP) and grape seeds (GS).

	Sample A	Sample B	Sample C
**Composition (v%)**	A0: 100% PBAT	B0: 50% PBAT, 50% PBS	C0: 100% PBS
	A1: 70% PBAT, 30% WL	B1: 30% PBAT, 30% PBS, 40% WL	C1: 70% PBS, 30% WL
	A2: 70% PBAT, 30% GP	B2: 30% PBAT, 30% PBS, 40% GP	C2: 70% PBS, 30% GP
	A3: 70% PBAT, 30% GS	B3: 30% PBAT, 30% PBS, 40% GS	C3: 70% PBS, 30% GS
	A4: 70% PBAT, 30% Ac. WL		C4: 70% PBS, 30% Ac. WL
	A5: 70% PBAT, 30% Ac. GS		C5: 70% PBS, 30% Ac. GS

**Table 3:** Granulometric data (D10, D50, D90) and real densities (ρ) of three different bio-fillers: wine lees (WL), grape pomace (GP) and grape seeds (GS).

Bio-Filler	D10 [μm]	D50 [μm]	D90 [μm]	ρ [g/cm^3^]
**WL**	7.7 ± 0.2	26.9 ± 1.5	149.7 ± 35.8	1.76
**GP**	65.3 ± 5.9	283.0 ± 5.7	496.5 ± 2.1	1.38
**GS**	60.9 ± 10.1	270.5 ± 9.2	491.0 ± 4.2	1.31

**Table 4:** Thermal characterization of three different bio-fillers: the not-volatile (*S_nv_*) and volatile (*S_v_*) solid fractions; the temperature corresponding to a mass loss of 10 wt. % (T_10wt. %_) and the residual mass after the thermogravimetric analysis at 900 °C.

Bio-Filler	*S_nv_* (%)	*S_v_* (%)	N_2_ Atmosphere		Air	
			T_10wt. %_ (°C)	Residual Mass (%)	T_10wt. %_ (°C)	Residual Mass (%)
**WL**	50.4	49.6	188	48	199	53
**GP**	6.6	93.4	232	20	243	7
**GS**	14.0	86.0	245	16	255	6

**Table 5:** PBAT and PBS-based samples’ thermal properties.

Code	*T_c_* (°C)	*H_c_* (J/g)	*T_g_* (°C)	*T_m_* (°C)	*H_m_* (J/g)	*X_c_* (%)	MFR (g/10 min)
**A0**	72.8	16.8	−37.9	119.8	10.6	9.3	5.2
**A1**	76.0	11.1	−38.4	118.2	4.6	5.8	10.9
**A2**	75.8	11.1	−37.5	119.5	6.0	7.5	7.0
**A3**	73.7	10.1	−38.9	119.6	6.7	8.4	9.1
**A4**	79.3	10.0	−38.6	121.1	5.4	6.7	11.4
**A5**	78.9	12.8	−39.1	121.3	7.3	9.2	9.8
**B0**	88.7	45.6	−38.7	113.6	45,6	40.7	5.6
**B1**	72.7	19.6	−36.6	110.8	17.2	42.5	11.1
**B2**	78.6	22.9	−38.9	112.1	20.8	51.5	10.1
**B3**	78.7	21.4	−37.8	112.4	20.5	50.9	8.8
**C0**	87.4	63.4	−38.4	114.4	63.7	57.9	5.7
**C1**	72.8	40.8	−38.4	112.1	40.8	53.6	9.3
**C2**	80.3	42.6	−38.0	113.6	41.3	53.6	8.3
**C3**	79.8	44.7	−38.4	113.3	41.6	54.1	8.9
**C4**	83.8	47.8	−39.1	113.4	45.6	59.3	10.4
**C5**	80.3	44.6	−39.1	113.2	44.8	58.1	9.2

**Table 6:** Results of tensile tests: Young’s modulus (E), ultimate tensile strength (UTS), and elongation at break (ε_b_).

Samples	E (MPa)	UTS (MPa)	ε_b_ (mm/mm)
**A0**	124 ± 5	17.5 ± 1.0	3.23 ± 0.65
**B0**	416 ± 11	24.5 ± 3.1	2.15 ± 0.22
**C0**	841 ± 29	39.4 ± 3.9	1.83 ± 0.13
**A4**	259 ± 10	12.1 ± 2.8	0.52 ± 0.09
**A5**	325 ± 2	13.7 ± 1.2	0.72 ± 0.03
**C4**	1381 ± 6	25.2 ± 0.4	0.11 ± 0.01
**C5**	1406 ± 22	25.4 ± 0.7	0.13 ± 0.01

**Table 7:** Storage moduli (E’) of bio-composites evaluated at different temperatures (0, 25, 50, and 75 °C).

Code	E’(0) (MPa)	E’(25) (MPa)	E’(50) (MPa)	E’(75) (MPa)
**A0**	92	53	21	10
**A1**	226	110	54	26
**A2**	180	119	55	29
**A3**	153	96	42	20
**A4**	331	222	113	52
**A5**	163	115	58	28
**B0**	163	115	58	28
**B1**	629	298	152	83
**B2**	269	183	100	55
**B3**	451	333	208	124
**C0**	588	450	330	222
**C1**	1045	630	41	252
**C2**	751	572	449	310
**C3**	741	601	417	278
**C4**	1040	827	627	460
**C5**	720	578	455	333

## Data Availability

The original contributions presented in the study are included in the article, and further inquiries can be directed towards the corresponding author.
